# Combined IVIG and High‐Dose Systemic Corticosteroids for the Management of Pembrolizumab‐Induced Cytokine Release Syndrome in Lung Adenocarcinoma

**DOI:** 10.1002/rcr2.70215

**Published:** 2025-05-19

**Authors:** Cheng‐Yin Liu, Chia‐Hsin Liu

**Affiliations:** ^1^ Division of Pulmonary and Critical Care Medicine, Department of Internal Medicine Tri‐Service General Hospital, National Defense Medical Center Taipei Taiwan; ^2^ Department of Internal Medicine Hualien Armed Forces General Hospital Hualien Taiwan

**Keywords:** cytokine release syndrome, intravenous immunoglobulin (IVIG), lung adenocarcinoma, pembrolizumab

## Abstract

We describe a case of a 61‐year‐old woman with stage IV lung adenocarcinoma (cT4N0M1a) and contralateral lung metastases who developed immune‐related adverse events (irAEs) following the 8th maintenance cycle of pembrolizumab and pemetrexed. Despite initiating systemic corticosteroids, her condition rapidly deteriorated into multiple organ dysfunction syndrome with elevated interleukin‐6 (IL‐6) levels. Tocilizumab was administered for suspected cytokine release syndrome (CRS) but resulted in minimal improvement. However, the subsequent administration of combined intravenous immunoglobulin (IVIG) and high‐dose systemic corticosteroids led to gradual recovery, and she was ultimately discharged in a stable condition.

## Graphical abstract

This report describes a rare case of pembrolizumab‐induced cytokine release syndrome (CRS) in a lung cancer patient, refractory to standard‐dose methylprednisolone and tocilizumab but successfully treated with high‐dose methylprednisolone and intravenous immunoglobulin (IVIG).

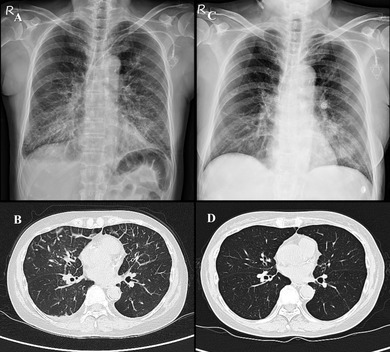

## 1 Introduction

Cytokine release syndrome (CRS) is a well‐known immune‐related adverse event, commonly linked to CAR T‐cell therapy but rarely reported with immune checkpoint inhibitors (ICIs) [1]. An analysis of the World Health Organisation's global pharmacovigilance database estimates the incidence of ICI‐related CRS at ~0.07% [2]. CRS triggers a systemic inflammatory response, potentially leading to multi‐organ failure. While IL‐6 inhibitors like tocilizumab are standard treatment, their effectiveness varies [3].

## 2 Case Report

A 61‐year‐old woman with a history of stage IV (T4N0M1a) lung adenocarcinoma harbouring a *TP53* mutation and programmed death‐ligand 1 (PD‐L1) expression (tumour proportion score [TPS] = 0) with contralateral lung metastases was initially treated with chemotherapy (carboplatin and pemetrexed) combined with pembrolizumab immunotherapy. After 6 months, a chest CT scan revealed a partial response of the lung masses, prompting a transition to triweekly maintenance therapy with pembrolizumab and pemetrexed.

**FIGURE 1:**
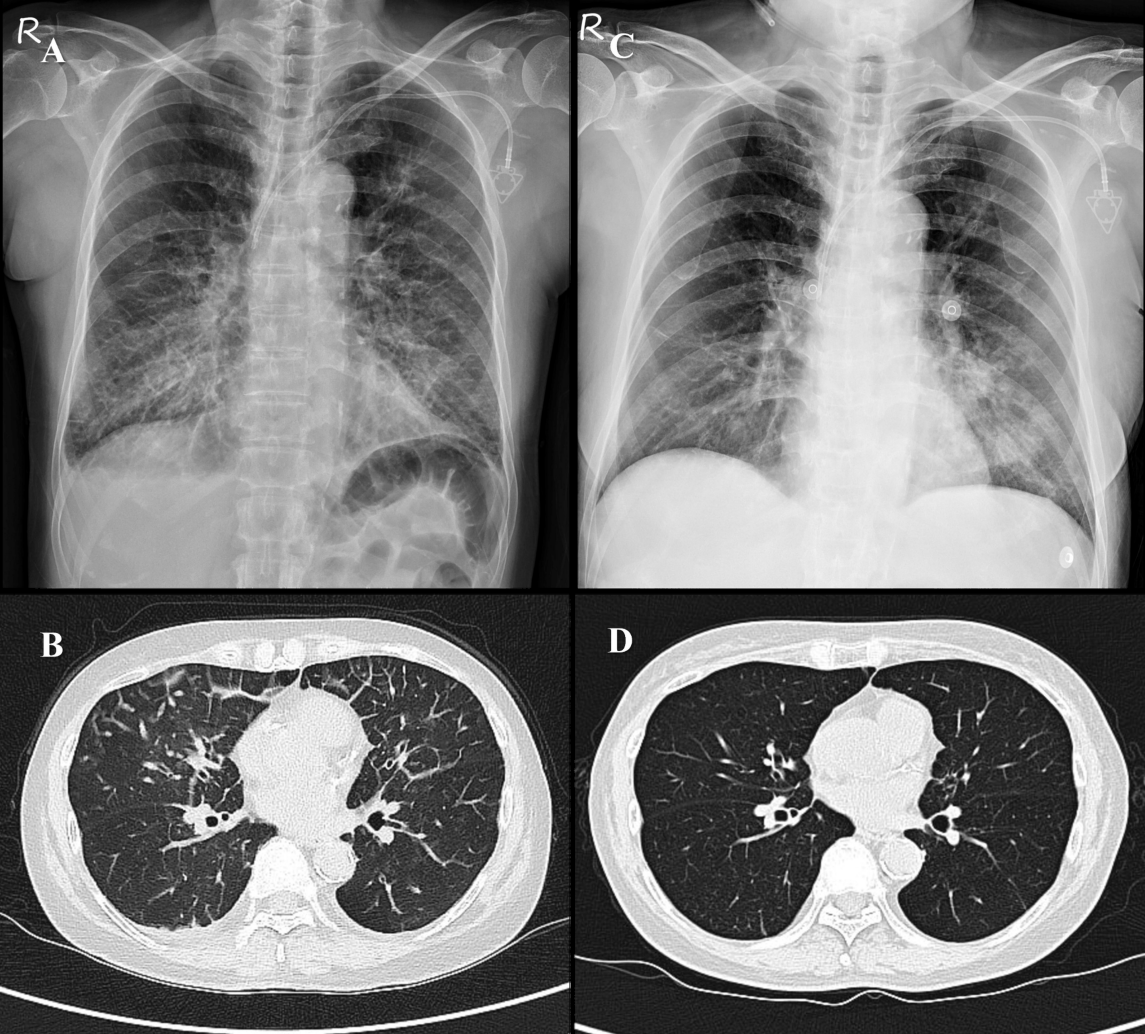
Radiographic findings before and after IVIG and high‐dose corticosteroid treatment. (A) Initial chest x‐ray showed diffuse lung opacities. (B) Baseline CT scan revealed bilateral interlobular septal thickening and ground‐glass opacities in both lungs. (C) Chest x‐ray on day 10 post‐treatment demonstrating significant resolution of opacities. (D) Follow‐up CT at 3 months revealed complete resolution of pulmonary infiltrates.

Following the eighth maintenance cycle, she developed a one‐week history of intermittent fever, myalgia and worsening exertional dyspnea. Physical examination revealed bilateral lung crackles. Laboratory investigations showed elevated C‐reactive protein (CRP) levels, and a chest x‐ray (CXR) revealed increased bilateral lung infiltrates (Figure 1A). Chest CT demonstrated stable pulmonary lesions but new interlobular septal thickening and ground‐glass opacities in both lungs (Figure 1B).

She was initially treated with systemic corticosteroids (methylprednisolone 40 mg every 12 h) for a presumed immune‐related adverse event (irAE) with interstitial lung disease, along with empiric antibiotics for suspected pneumonia with sepsis. Prior to initiating immunosuppressive therapy, a comprehensive microbiological workup was performed, including bacterial cultures (blood and sputum), respiratory viral PCR panel and Pneumocystis jirovecii PCR, all of which returned negative results.

**FIGURE 2:**
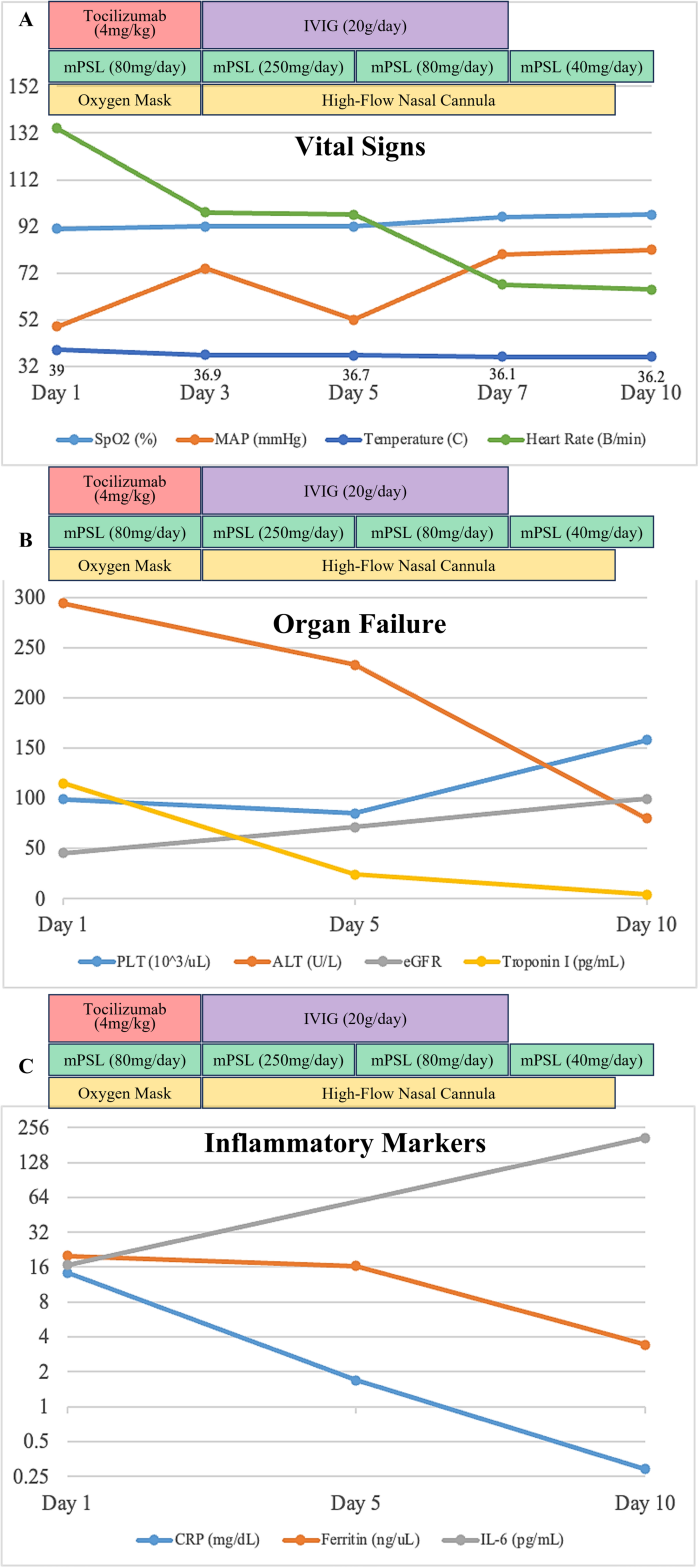
Clinical parameters during CRS management. (A) Trends in SpO_2_, MAP, body temperature and heart rate over 10 days. Oxygen therapy and interventions (tocilizumab, IVIG and corticosteroids) are indicated. (B) Organ dysfunction markers (PLT, ALT, eGFR, troponin I) showed improvement after IVIG and high‐dose corticosteroids. (C) Changes in inflammatory markers (CRP, ferritin, IL‐6) reflecting treatment response. IVIG, intravenous immunoglobulin; MAP, mean arterial pressure; mPSL, methylprednisolone.

Despite treatment, her condition deteriorated, progressing to multiple organ dysfunction syndrome characterised by hypotension, hypoxemic respiratory failure, renal failure, thrombocytopenia, and acute liver injury, accompanied by elevated interleukin‐6 (IL‐6) and ferritin levels (Figure 2). Given the suspicion of cytokine release syndrome (CRS), tocilizumab (200 mg/day) was administered for 2 days but showed limited efficacy (Figure 2). Her respiratory status worsened, necessitating escalation from oxygen mask therapy to a high‐flow nasal cannula (HFNC). Due to her worsening condition, high‐dose glucocorticoids (methylprednisolone 250 mg/day for 2 days) were initiated in combination with intravenous immunoglobulin (IVIG, 20 g/day for 5 days) (Figure 2).

A follow‐up chest x‐ray (CXR) on day 10 post‐treatment showed significant resolution of bilateral lung opacities (Figure 1C). As her symptoms improved, systemic corticosteroids were gradually tapered and transitioned to oral prednisolone. She was eventually discharged in stable condition. A follow‐up CT at 3 months confirmed the complete resolution of lung lesions (Figure 1D).

## 3 Discussion

Immune checkpoint inhibitors have revolutionised advanced NSCLC treatment, but irAEs can be severe. Cytokine release syndrome (CRS), a rare yet serious irAE, results from excessive immune activation. This report describes a rare case of pembrolizumab‐induced CRS in a lung cancer patient, refractory to standard‐dose methylprednisolone and tocilizumab but successfully treated with high‐dose methylprednisolone and IVIG.

Pembrolizumab was used in this case despite a PD‐L1 TPS of 0 because the KEYNOTE‐189 study demonstrated clinical benefit in patients receiving pembrolizumab combined with chemotherapy, regardless of PD‐L1 expression levels. Specifically, even patients with PD‐L1 TPS < 1% (including TPS = 0) showed significantly improved overall survival and progression‐free survival compared to chemotherapy alone [4]. Thus, the use of pembrolizumab in combination with chemotherapy is justified by these data.

Diagnosing ICI‐related CRS is challenging due to its overlapping features with irAEs and sepsis. Our patient initially presented with fever, dyspnea, elevated inflammatory markers (CRP, ferritin) and increased lung infiltrates, raising concerns for pneumonitis or pneumonia. The progression to multi‐organ failure, along with markedly elevated IL‐6 levels and the absence of a confirmed infection, further supported an immune‐mediated process, confirming the diagnosis of CRS. Although distinguishing the clinical symptoms of CRS from those of sepsis can be challenging, pre‐emptive broad‐spectrum antibiotics may be recommended following appropriate infection testing.

Tocilizumab, an IL‐6 inhibitor, blocks the IL‐6 receptor, leading to transient elevations in serum IL‐6 levels. While high IL‐6 levels are typical in severe CRS, distinguishing between receptor blockade effects and disease progression remains challenging [5]. Tocilizumab is the standard treatment for CRS in CAR T‐cell therapy, but its effectiveness in ICI‐induced CRS is unclear [3]. Tocilizumab was administered at a lower dose of 200 mg per day due to concerns about the patient's frail condition, renal function, and the potential for severe immunosuppression. Given the absence of strong clinical guidelines for dose adjustments in such scenarios, the treating team opted for a cautious approach. However, the patient showed minimal improvement despite two doses, suggesting that additional inflammatory pathways might be involved. In such cases, high‐dose methylprednisolone may be considered post‐IL‐6 inhibition to help mitigate systemic inflammation and reduce the risk of multi‐organ failure.

Given the immune hyperactivation in CRS, IVIG was used as adjunctive therapy to suppress inflammation, neutralise cytokines and provide passive immunity against occult infections [6]. This is especially relevant in immunosuppressed patients at risk for sepsis after IL‐6 inhibitors and high‐dose steroids. IVIG's immunomodulatory effects, including Fc receptor inhibition and cytokine suppression, likely contributed to our patient's recovery. Its efficacy in steroid‐refractory irAEs and systemic inflammatory syndromes supports its potential role in severe ICI‐related CRS. In our patient, IVIG and high‐dose methylprednisolone led to significant clinical improvement, highlighting their therapeutic value.

CRS is a rare but serious complication of ICI therapy in NSCLC, with no standardised treatment guidelines. This case highlights the importance of early recognition and intervention in ICI‐treated patients presenting with systemic inflammation and organ dysfunction. While tocilizumab is a standard treatment, it may not always be effective. Our case demonstrates that when standard‐dose corticosteroids and tocilizumab fail, high‐dose corticosteroids and IVIG can serve as a safe and effective alternative.

## Author Contributions

Cheng‐Yin Liu and Chia‐Hsin Liu wrote the manuscript. Cheng‐Yin Liu and Chia‐Hsin Liu performed collection, analysis and interpretation of data.

## Consent

The authors declare that written informed consent was obtained for the publication of this manuscript and accompanying images using the consent form provided by the Journal.

## Conflicts of Interest

The authors declare no conflicts of interest.

## Data Availability

Data sharing is not applicable to this article as no new data were created or analyzed in this study.
